# Neonatal past catches up when COVID-19 comes to town

**DOI:** 10.1038/s41390-024-03237-w

**Published:** 2024-05-07

**Authors:** Georgie Dowse, David G. Tingay, Julia Charlton

**Affiliations:** 1 Neonatal Research, Murdoch Children’s Research Institute, Melbourne, VIC Australia; 2 Department of Paediatrics, University of Melbourne, Melbourne, VIC Australia; 3 Department of Pediatrics, University of British Columbia, Vancouver, BC Canada; 4 Division of Neonatology, BC Women’s and Children’s Hospital and Health Centre, Vancouver, BC Canada

Congenital anomalies are structural or functional changes that are present at birth. Whilst each is rare, together they are common, occurring in one in every 33 babies born in the United States.^1^ Importantly, congenital anomalies remain a leading cause of severe childhood mortality, morbidity, and disability, especially in infancy. In adults, the presence of underlying medical conditions is known to increase the risk of severe illness from COVID-19,^2^ but less is known about the risk factors in children. This is unfortunate as the impact of severe illness in children can be long-lasting. Congenital heart disease has been shown to lead to more severe COVID-19 outcomes.^3^ Whether other congenital anomalies predispose children to severe COVID-19 remains less well-understood, but the higher rates of severe illness from other common childhood infections, such as respiratory syncytial virus,^4^ prompt further reflection.

In this issue of *Pediatric Research*, Goodman et al. conducted a retrospective, observational cohort study to explore the relationship between a history of congenital anomalies and the severity of COVID-19 in paediatric patients.^5^ The authors utilised a large database of electronic health records (Cerner Real-World Data) to investigate 927,805 paediatrics patient hospital-based encounters with COVID-19 from 117 health systems across the United States between March 2020 to February 2022. Congenital anomalies were classified using the International Statistical Classification of Disease Codes (ICD-10-CM) and divided into 10 groups based on the system. COVID-19 severity was defined by the level of oxygen and respiratory support delivered, consistent with previous guidelines.^6^ A strength of the study was that cases were matched with children without a documented congenital anomaly who presented with SARS-CoV-2 infection using 1:1 propensity scores on age, sex, race, chronic illness (including obesity), healthcare funder, and time point within the pandemic.

The authors identified that all categories of congenital anomalies (106,980 children) were associated with more severe COVID-19 than those without a history of congenital anomalies, with cardiovascular anomalies conferring the greatest risk. The increased odds of experiencing severe COVID-19 in children ranged from 1.16 [95% CI 1.03, 1.31] for congenital head and neck anomalies to 3.84 [3.63, 4.06] for cardiovascular anomalies. It is not surprising that cardiovascular anomalies were most strongly associated with severe COVID-19, as pre-existing cardiac disease is a known risk factor for severe COVID-19 in adults.^7^ Cardiovascular anomalies in children are often associated with immune deficiencies and usually require surgery. In many cases, the surgery is not curative, and children experience lifelong cardiac and neurodevelopmental sequelae. However, the reasons for the higher association with severe COVID-19 found in children with other types of anomalies, such as cleft lip/palate (2.87 [2.48, 3.32]) are less obvious, particularly as the odds were higher in these conditions than for respiratory conditions (2.24 [2.04, 2.46]). Whether this group of children had completed surgical repair of the cleft or not was not reported; categorisation of congenital anomalies was based on the initial structural findings rather than treatments and functional implications. Interestingly, a recent population-based study in Japan highlighted an increased incidence of lower respiratory tract infections during the first year of life in infants with orofacial clefts (when most cleft palates would not be completely repaired) compared with a control group (risk ratio 2.73 [95% CI 1.40, 5.33] for cleft lip) after adjusting for covariates.^8^

Causal relationships can only be correctly interpreted following identification and appropriate adjustment of confounders and potential sources of bias.^9^ Congenital anomalies often occur within a constellation of genetic contributors, other anomalies, comorbidities, and/or complications of early-life treatments. The biological interactions between these factors are not known in this study, but it is likely that these factors play a role in causing congenital malformations, as well as altering the immune response to increase the risk of severe disease. The most compelling potential confounder was unfortunately not accounted for in the analyses; that of gestational age at birth. There is a well-described link between prematurity and congenital malformations,^10,11^ and there is an increased risk of longer-term sequelae when the two co-exist compared with each in isolation. Interestingly, congenital pulmonary anomalies are the only group of conditions that are not associated with an increased risk of prematurity and are the only conditions that do not occur more frequently in preterm infants than in term-born infants.^12^ This may explain why underlying respiratory anomalies are not as prominent a risk factor for severe COVID-19 disease in this study. Similarly, the secondary functional impacts of some anomalies may influence infection risk and interpretation of disease severity, such as reduced respiratory capacity, impaired bulbar function, relatively short bowel, and reduced cardiac reserve. The consistent finding across all congenital anomaly groups of increased severity risk highlights the need to consider these findings as associated rather than causal. In isolation, each group of congenital anomalies is relatively rare, making risk analysis subject to skew from information bias. It is only through larger, population-based studies, such as this conducted by Goodman et al. that important associations can be identified.^5^ This highlights how important it is to find methods of studying rare and complex conditions over the longer term if the goal is to minimise the health burden on children and families who already likely have increased healthcare needs.

Selection bias is a possibility in this study, as the study population only included children presenting to a hospital emergency department or admitted to a hospital and cannot be considered representative of the true population. In general, children are known to have a lower risk of severe illness from SARS-CoV-2 infection compared to adults. Thus, 20% of children developing moderate or severe COVID-19 is unexpected and significantly higher than other reports.^13,14^ Children discharged from NICUs with complex medical needs have high rates of repeat admissions to hospitals, including intensive care.^15^ More than 10% of infants discharged from NICUs with significant complex care needs are those with congenital anomalies,^15^ so it is not surprising that there would overlap with this categorisation of infants. It could also be postulated that parents of a child with congenital anomalies, are more likely to present to a hospital setting than those without underlying medical conditions. In all, this suggests that congenital anomalies managed as neonates leave a legacy that appears to increase the subsequent severity of illness when exposed to infections like COVID-19 later in life.

In addition to this selection bias, healthcare workers may also apply a different prism, with different thresholds to admit or begin escalating treatments in children with a background of congenital anomalies. The inability to generate high-quality evidence for treatments in children remains frustrating, especially in rare diseases, where large interventional trials are often not feasible. The impact of this was evident during the COVID-19 pandemic. The large trial networks that rapidly generated evidence for existing and novel treatments rarely included children. Consequently, most COVID-19 treatment guidelines for children are more cautious than for adults.^6^ That corticosteroids and heparin, both treatments with an established familiarity in paediatric critical care, were the most used treatments in children developing severe COVID-19 in this study, may reflect this inequity in healthcare research. The authors should be congratulated for utilising a large, EMR-derived database to identify a high-risk group of paediatric patients who require greater clinical attention when presenting with COVID-19. Such databases are arguably a pragmatic solution that should be used more in paediatric research, providing rigorous mechanisms are in place to ensure the data is of high quality, accurate and all potential types of confounders considered.

This observational study was also limited to health systems in the United States. The results may not reflect populations in other countries, lacking external validity due to differences in resources, practices, availability of vaccines, and public health restrictions. The study period included the Alpha, Delta, and Omicron variants of SARS-CoV-2. SARS-CoV-2 variant and vaccination status were not controlled for. However, the potential influence of these confounders was hopefully reduced by matching patients on the month of encounter.

SARS-CoV-2 infection has become one of many infectious illnesses common in children. This study is a timely reminder that early life events confer a greater risk for common illnesses in childhood and being born with a congenital anomaly can carry a long-lasting legacy.
